# Drug_SNSMiner: standard pharmacovigilance pipeline for detection of adverse drug reaction using SNS data

**DOI:** 10.1038/s41598-023-28912-6

**Published:** 2023-03-07

**Authors:** Seunghee Lee, Hyekyung Woo, Chung Chun Lee, Gyeongmin Kim, Jong-Yeup Kim, Suehyun Lee

**Affiliations:** 1 Healthcare Data Science Center, Konyang University Hospital, Daejeon, 35365 Republic of Korea; 2 Department of Health Administration, Kongju National University, Gongju, 32588 Republic of Korea; 3 Department of Biomedical Informatics, College of Medicine, Konyang University, Daejeon, 35365 Republic of Korea; 4 Department of Biomedical Engineering, Konyang University, Daejeon, 35365 Republic of Korea; 5 Institute of Health and Environment, Kongju National University, Gongju, 32588 Republic of Korea; 6 College of IT Convergence, Gachon University, Seongnam, 13120 Republic of Korea

**Keywords:** Health care, Medical research

## Abstract

As society continues to age, it is becoming increasingly important to monitor drug use in the elderly. Social media data have been used for monitoring adverse drug reactions. The aim of this study was to determine whether social network studies (SNS) are useful sources of drug side effects information. We propose a method for utilizing SNS data to plot the known side effects of geriatric drugs in a dosing map. We developed a lexicon of drug terms associated with side effects and mapped patterns from social media data. We confirmed that well-known side effects may be obtained by utilizing SNS data. Based on these results, we propose a pharmacovigilance pipeline that can be extended to unknown side effects. We propose the standard analysis pipeline Drug_SNSMiner for monitoring side effects using SNS data and evaluated it as a drug prescription platform for the elderly. We confirmed that side effects may be monitored from the consumer’s perspective based on SNS data using only drug information. SNS data were deemed good sources of information to determine ADRs and obtain other complementary data. We established that these learning data are invaluable for AI requiring the acquisition of ADR posts on efficacious drugs.

## Introduction

Adverse drug reactions (ADRs) are a major public health problem for the aged. As the number and variety of approved drugs increase, it is vital to assess the effects of these medications on the patient population at large. To this end, information must be collected and data analyses must be performed. Thus, it is critical to monitor the safety of drugs already launched on the market^1–3^. The International Conference on Harmonization considers older people a ‘special population’ as they differ from younger adults in terms of comorbidity, polypharmacy, pharmacokinetics, and vulnerability to ADRs^4,5^. As society continues to age, it is increasingly important to monitor drug use in the elderly.

The spontaneous reporting system is a widely used, effective, and relatively inexpensive method of collecting information on suspected ADRs. Its main function is to detect new, rare, and serious ADRs that were overlooked in pre-marketing clinical trials. Spontaneous reporting is applied from the day a drug is first launched and throughout its market life. The spontaneous reporting system provides information gleaned from real-life clinical practice rather than clinical trials. In the latter case, vulnerable individuals are excluded and the treatment duration is short. However, the spontaneous reporting system has several shortcomings such as underreporting^6,7,30^. Current spontaneous reporting systems and pharmacovigilance may be enhanced by using expanded data sources, including those available on social media sites such as Twitter and on health-related social networks such as DailyStrength^8,9,31^.

On social media, patients write about medications they have taken and the ADRs they believe that they might have experienced because of these drugs. Social media data were proposed as an auxiliary method of monitoring ADRs^10,11^. Causal relationships have been assessed by analyzing ADR social network study (SNS) data and online meetings of patients with the same diseases^12,13^. As the personalized healthcare industry is revitalized, however, there is growing interest in the provision of pharmaceutical information services using healthcare data. Along with structured data, such as patient medical records, unstructured text data are also being considered such as expert medical opinions, ADR information posted by individuals on social media, and published research results^14–19^.

The aim of this study was to determine whether social network studies are useful as sources of information for monitoring drug side effects. We propose a method for exploring known side effects of geriatric drugs in a dosing map by utilizing SNS data. We selected and analyzed the drugs most frequently prescribed for the elderly at Konyang University Hospital, South Korea. To this end, we implemented the standard drug analysis pipeline Drug_SNSMiner for drug safety verification based on the SNS data. We tested the protocol with ketoprofen, which is a frequently prescribed geriatric drug. Each step in the analysis was systematically modularized so that a continuous pipeline could be developed and applied to other drugs and side effects.

## Methods

### SNS data-based standard analytical pipeline: drug_SNSMiner

**Figure 1:**
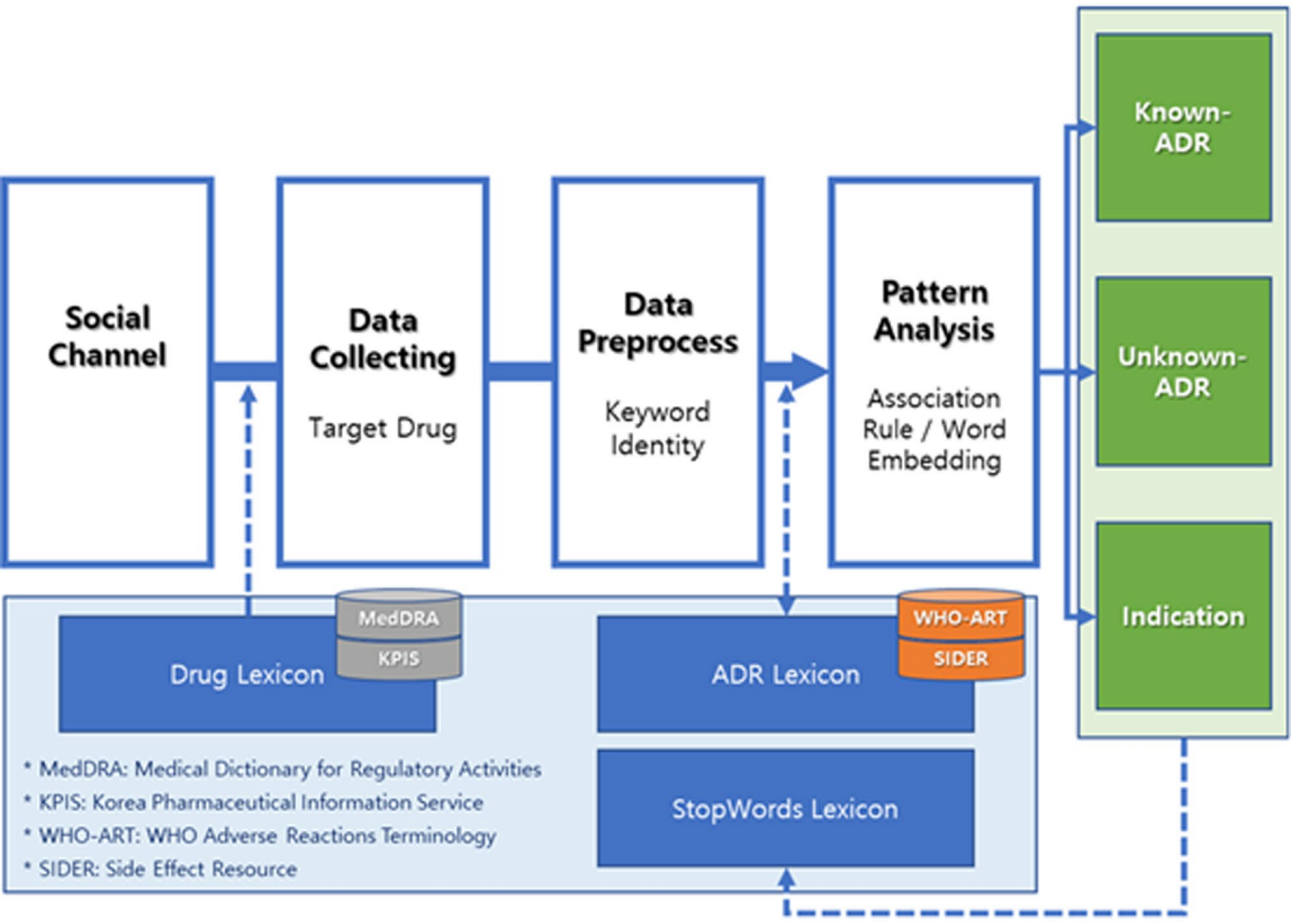
Overview of Drug_SNSMiner pipeline steps and study design.

A standard analytical pipeline was proposed for pharmacovigilance based on SNS data. It was named Drug_SNSMiner and its procedural steps are summarized as follows. Data were procured by selecting appropriate social channels for the pharmacovigilance targets and by defining lexicons for the drugs to be analyzed. For the data acquired, the Lexicon was extracted and defined based on side effects related to the target drugs. The latter were mapped from a standard drug database, and the lexicon of stop words was prepared and supplemented for text preprocessing. In this manner, drug and side effect patterns could be elucidated, social postings for known side effects could be identified, and novel candidates for unknown side effects and indications could be investigated (Fig. 1).

An analysis was performed on the geriatric drugs frequently prescribed at Konyang University Hospital and listed on Naver, which is the largest social channel in South Korea. According to Drug_SNSMiner, drug side effect information could be gathered using SNS data. Three lexicons (Drug, ADR, and StopWords) were implemented to obtain relevant posts and the pattern analysis results were used to classify them based on side effects lexicons. Medical Dictionary for Regulatory Activities (MedDRA) Preferred Term was used as a mapping key to define the World Health Organization Adverse Reactions Terminology (WHO–ART) and Side Effect Resource (SIDER)-based lexicons of well-known side effects. The complement of known adverse events in the WHO-ART adverse event terminology database may be naturally defined as unknown adverse events. It was expected that it could be extended to additional pattern analyses in the same context.

### Lexicon definitions

1) Drug

The drug term lexicon was based on the drug code provided by the Korea Pharmaceutical Information Service (KPIS). A prior study^20^ calculated the Beers Criteria drug prescription and side effects incidences in persons aged ≥ 65 years in South Korea. The top three drugs (metoclopramide, chlorpheniramine, and ketoprofen) were selected based on prescriptions and side effects reported for the clinical environment of Konyang University Hospital. Here, the focus was directed to ketoprofen as it had the most abundant data.

2) ADR

WHO–ART is a dictionary used for rational coding of adverse reaction terms. The system was maintained by the Uppsala Monitoring Centre (UMC) and the World Health Organization Collaborating Center for International Drug Monitoring, but it is no longer actively maintained^21^. SIDER is a datasbase that records side effect information for marketed drugs. The names of the drugs provided by SIDER are based on the FDA drug label. The names of the side effects are terms in MedDRA^22^. In the present study, custom side effect words were added for each target drug.

3) StopWords

In computing, stop words are filtered out before and/or after natural language data processing. A custom lexicon was added to the basic stop words dictionary comprising 677 entries (https://www.ranks.nl/stopwords/korean). Other user-defined stop words were added as well. A total of 7,195 stop words dictionaries were created and the data were preprocessed with them.

### Data collection

The use of social big data as a new data resource is being investigated in various fields. At online cafes, vast amounts of text data are generated in real time and they address various social issues. At online cafes and in blogs, numerous individuals gather to share interests, form relationships, and exchange information and opinions. Hence, new users are continuously attracted. In this study, Naver was selected as the channel and data source as it is the largest online community in South Korea.

**Figure 2:**
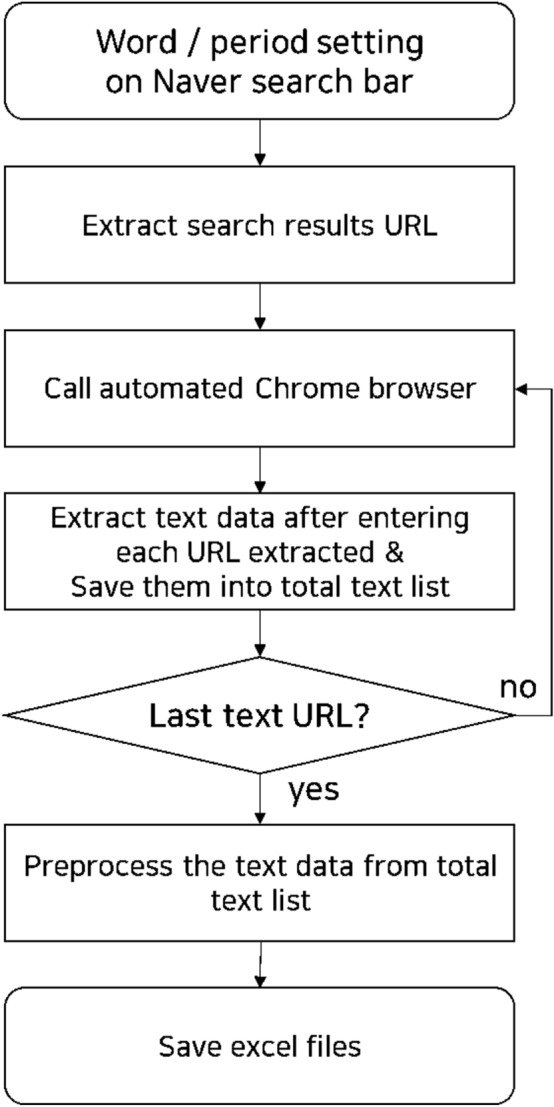
Data collection process flow.

There is a limit to the number of posts that can be consulted in the process of curating data with Naver Open API. Page number information for the results was obtained by entering a search query in the cafe and blog search platforms. URLs of the posts from the page were recorded and only the post body content was collected. Sensitive data such as ID and cafe name were excluded (Fig. 2).

The cafes and blogs of Naver were crawled based on the drug term lexicon. Posts were extracted such that > 10 posts using the same drug name were queried per ingredient. E-mail addresses, URLs, html tags, \r, \n, and special symbols were removed and only Hangul was extracted. Double spaces, and Hangul consonants and vowels used alone were also excluded.

### Data preprocessing

For rapid communication and convenience, spaces between words are seldom used in SNS posts. However, spacing errors between words can mar tokenization and part-of-speech tagging. Therefore, space preprocessing is required before tokenization to optimize natural language processing performance.

Soyspacing was used as a preprocessing model in word spacing. As it has an algorithm that learns spacing patterns from data, it is difficult to apply to a wide range of sentences. Nevertheless, it is suitable for specific domain data such as news articles and dialogues. However, if soyspacing does not appear several times in the training data, spacing may be applied incorrectly. To reduce these errors, additional spacing rules must be applied. Here, drug names were added to the rule dictionary so that words were not spaced.

MeCab (Kudo, 2006) is an open-source morphological analyzer based on Conditional Random Fields^23^. Mecab-ko-dic is a Korean morpheme analyzer using Mecab, which is an open-source morpheme analysis engine^24^. In the present study, word tokenization was performed using the "Eunjeon Han" morpheme analyzer in the corpus that was preprocessed by soyspacing.

The nouns extracted by tokenization include those that were irrelevant to this research. Such nouns degrade natural language processing performance. To conserve only meaningful nouns, extraneous words were selected in the study design, added to the stop word list, and removed.

Kiwi (Korean Intelligent Word Identifier) is a South Korean stemming analyzer library designed for high-speed, universal performance. It is highly useful as it provides additional functions such as unregistered word extraction. It is being released in open-source format so that anyone interested in South Korean natural language processing can readily apply it^25^.

### Pattern analysis

Association analysis is frequently used in data mining. It determines whether dichotomous variables (items) frequently appear together in a database. Association analysis detects groups with variables that are highly correlated to each other or to specific targets^28^. A chord diagram increases the abstraction level in visualizing relationships among network nodes. It is widely used in many disciplines to investigate patterns in social, biological, and other networks^21^.

Word2vec was used to extract words near ‘side effects’ after word embedding. Word embedding expresses words as dense vectors and word2vec embeds words. Word2vec is trained to use a distributed representation for words that frequently appear close together in the corpus data and within a close vector space. Word2vec overcomes the limitation of sparse representations that cannot express word similarity. A sparse representation separately expresses a word within a high-dimensional space. Hence, similarities between words can be calculated because other words for which similarity cannot be determined are distributed and expressed in the representation space^11^. Analyses were computed with R v. 3.6.3 for Windows (64-bit). R is a free software environment for statistical computing (R Core Team, Vienna, Austria; https://www.r-project.org).

## Results

### Data

**Figure 3:**
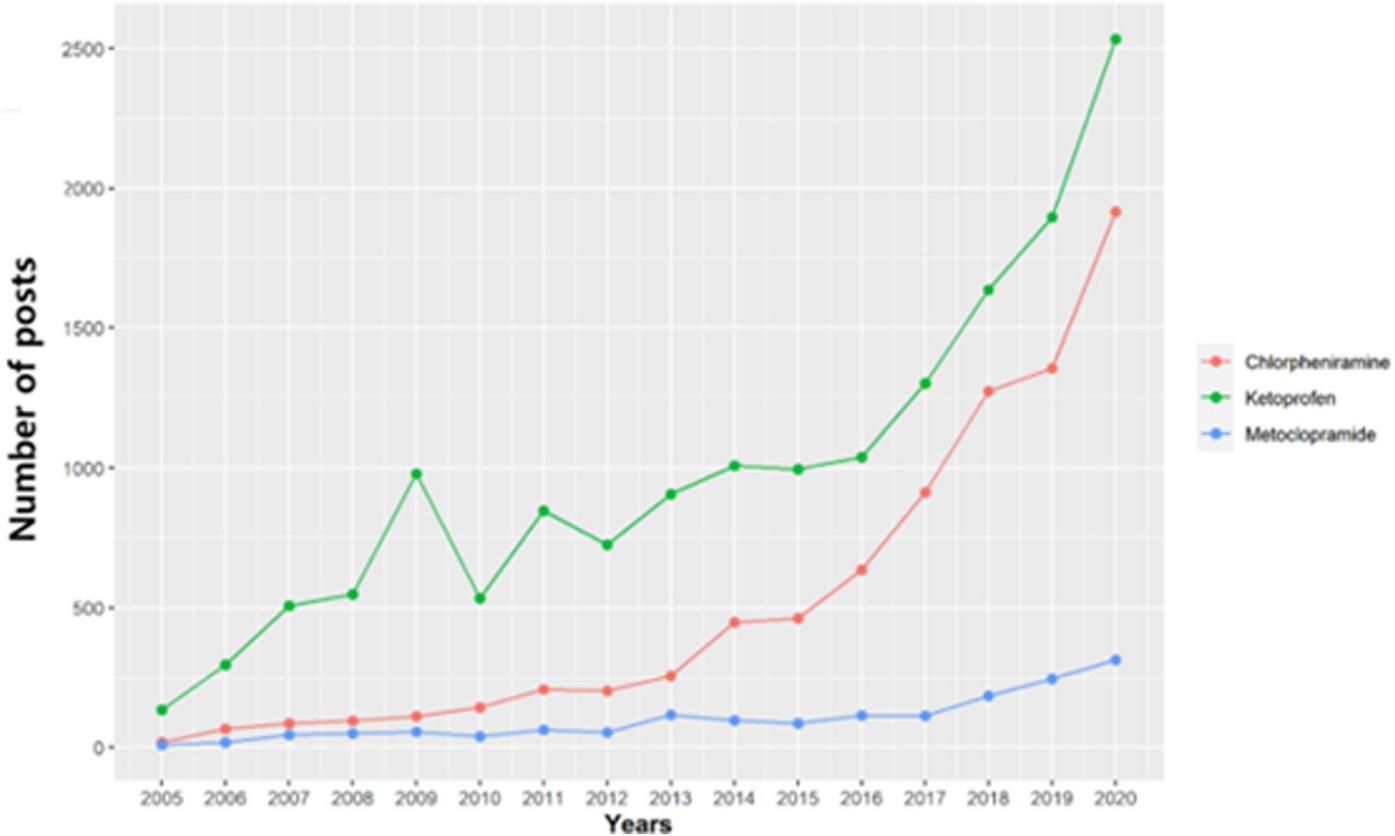
Trends analysis. Curation of articles mentioning each target drug.

Web crawling compiled 25,693 posts from 2005 to 2020. Ketoprofen increased the number of posts ≥ 18 × while chlorpheniramine increased the number of posts ≥ 100 × relative to 2005. Over time, individuals have been more actively sharing drug information via social media (Fig. 3). Since 2014, Chlorpheniramine posts have been steadily increasing and a growing number of general cold and nasal congestion medicines containing this ingredient have been marketed. Chlorpheniramine was approved and marketed in March 2014. Ketoprofen posts reached a local maximum in 2009 and have been steadily increasing since 2014. A blog-based ketoprofen promotional event in 2009 induced many posts. Since 2014, Antiphlamine Coin Plaster and other products have been continuously released. Antipuramine-related posts have increased. Fastum Gel and Antiphlamine Double Power Cataplasma were approved in October 2008 and marketed in August 2014.

We conducted an analysis of ketoprofen as it could secure the most posts. We acquired 14,156 crawled posts on ketoprofen from Naver blogs and cafes. We removed the advertisements and refined the lists to 6,232 posts. We obtained 5,126 posts containing the drug name at least once. Of these, 3,828 posts containing a word in the side effects dictionary were extracted at least once and 3,591 posts and 45,140 words were obtained after duplicate posts were removed.

After word clouding for the nouns extracted after ketoprofen tokenization, ‘Ketotop’ and ‘antipuramine’ took the first place as these ketoprofen-based anti-inflammatory analgesics are widely used in Korea. They were followed by painkiller-related words.

### Lexicon: drug, ADR, Stopwords

The search query for each drug was as follows: Metoclopramide (Metoclopramide, Mexolon, Macperan Tab, Meccol Injection), Chlorpheniramine (Chlorpheniramine, Peniramin Injection, Peniramin Tab, Peniramin), and Ketoprofen (Ketoprofen, Rheutin Cap, Cyprogel Ointment 3%, Ketotop, Kenofen Gel, Kefentech Plaster, Ketotop Gel, Fastum Gel, Antiphlamine Pain Relieving Roll Patch, Antiphlamine).

We used information from WHO–ART and SIDER as they are well-known standard databases for creating ADR lexicons. Both resources were consistent with MedDRA Preferred Terms. We then added real-time terms that were discovered as consumer words on social media. This task captured pertinent consumer words. For 'ketoprofen', the phrase 'something comes up' describes a skin disease. We divided the ketoprofen ADR lexicon into 29 SOC categories, including consumer words and 1,816 ADR words.

In this study, stop words were removed in two stages. First, those not normally used were identified and removed. The list of 904 stop words included unnecessary conjunctions, prepositions, verbs, and adjectives. After stop words processing, the number of nouns was reduced from 46,686 to 46,487. Non-drug terms were also identified and removed. The non-drug list included 822 stop words. After processing, 45,667 words had been collected (Table 1).

**Table 1:** Sample terms from each lexicon in Drug_SNSMiner.

	Number of terms	Sample terms lists
Drug lexicon	9	Ketoprofen, Rheutin Cap, Cyprogel Ointment 3%, Ketotop, Kenofen Gel, Kefentech Plaster, Ketotop Gel, Fastum Gel, Antiphlamine
ADR lexicon	1,816	Rashes, Edema, Hypersensitivity, Spasm, Anxiety, Swelling, Dizziness, Dry, Photosensitivity, Flushing, etc
Stop words lexicon	45,667	On the contrary, everyone, moreover, as much as possible, so, barely, on the occasion of, that much, the rest, of them, therefore, first of all, okay, just like, just like, etc

### Detected ADR words

1) ADR words derived from association analysis

We visualized the results of the correlation analysis with improvement ≥ 1.0, reliability ≥ 0.6, and support ≥ 0.015. For the degree of red coloration, the improvement was ~ 2 when the color was deep and ~ 1 when it was shallow. In general, when the improvement was ≥ 1, there were positive relationships between words. Reliability refers to the number of posts wherein specific words simultaneously appear with respect to the number of posts containing a specific word.

We visualized a co-occurrence frequency of > 60% for a specific rule. Support is the number of simultaneous occurrences of two words in all posts. Posts referring to drugs and side effects could not be ruled out as even a single appearance could be meaningful. Thus, we set the support level as low as possible to generate multiple rules. We examined the associations between the words in the side effects dictionary and the drug names. For 1,272 association rules, we identified 348 and 829 words related to the side effects of antipuramine and ketoprofen, respectively.

**Figure 4:**
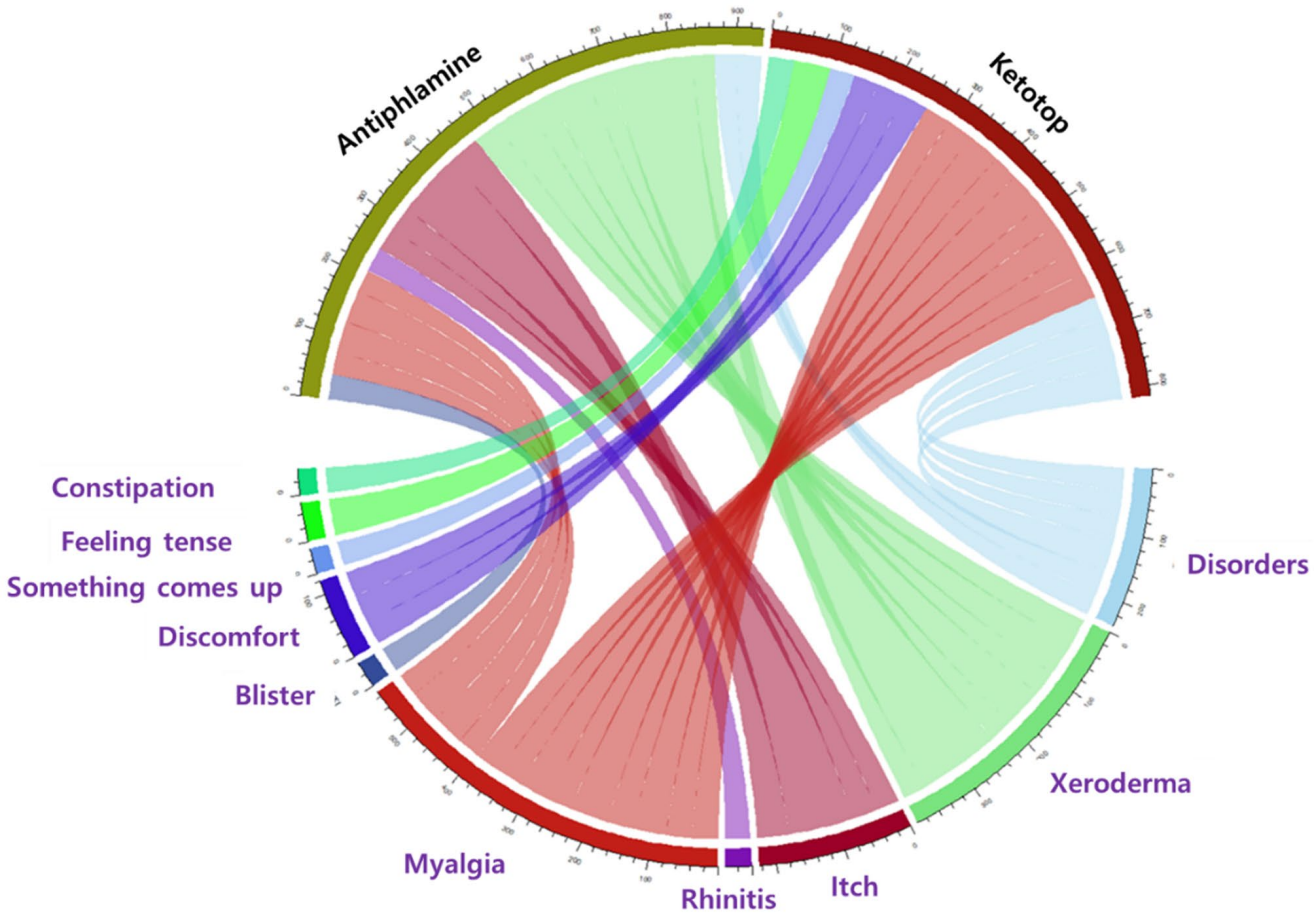
Chord diagram and graph for ketoprofen and antipuramine. Black letters indicate drug name searches and purple letters indicate drug-related side effect searches.

Figure 4 is a chord diagram of the relationship between drugs and side effects. A corpus was created by removing stop words from the words extracted from the crawled posts. We set the support level and reliability to 0.01 and 0.6, respectively, and performed a correlation analysis between drug names and side effects words for the entire corpus. The upper and lower parts of the diagram are the drug names and main drug side effects, respectively. For ketoprofen or a representative drug containing it, the most frequent association was muscle pain with dryness. For antipuramine or a representative drug containing it, the most frequent association was dryness.

2) ADR words derived by word2vec

**Figure 5:**
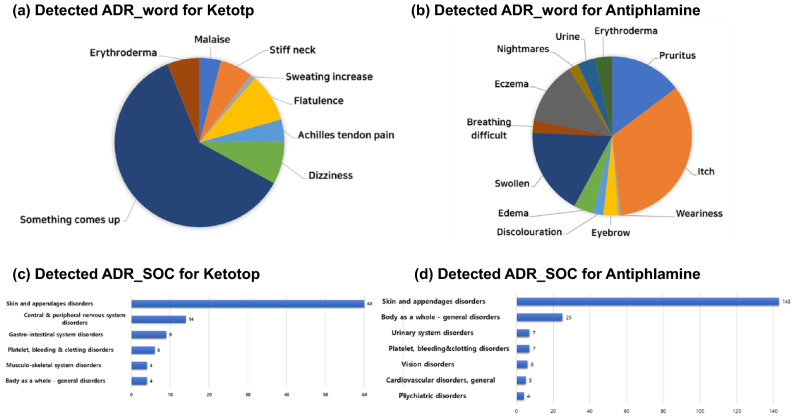
Graphs of each detected ADR word and SOC distribution for ketoprofen and antipuramine, which were the major drug names in the Drug Lexicon.

We used a skip-gram-based word2vec model and its 300-dimensional window size was 10. The diagram in Fig. 5 shows the results of counting side effects-related words extracted from crawled posts using a drug name as a search keyword. In posts crawled with ketoprofen as a search keyword, the leading words included oar (going up) and dizziness. In posts crawled with antipuramine as a search keyword, the leading words were itching, swelling, and so on. The bar graph was plotted by mapping to the SOC of MedDRA all side effects words extracted by the drug and counting them. For ketoprofen, skin and appendages disorders prevailed whereas for antipuramine, appendages disorders predominated.

### External validation

**Figure 6:**
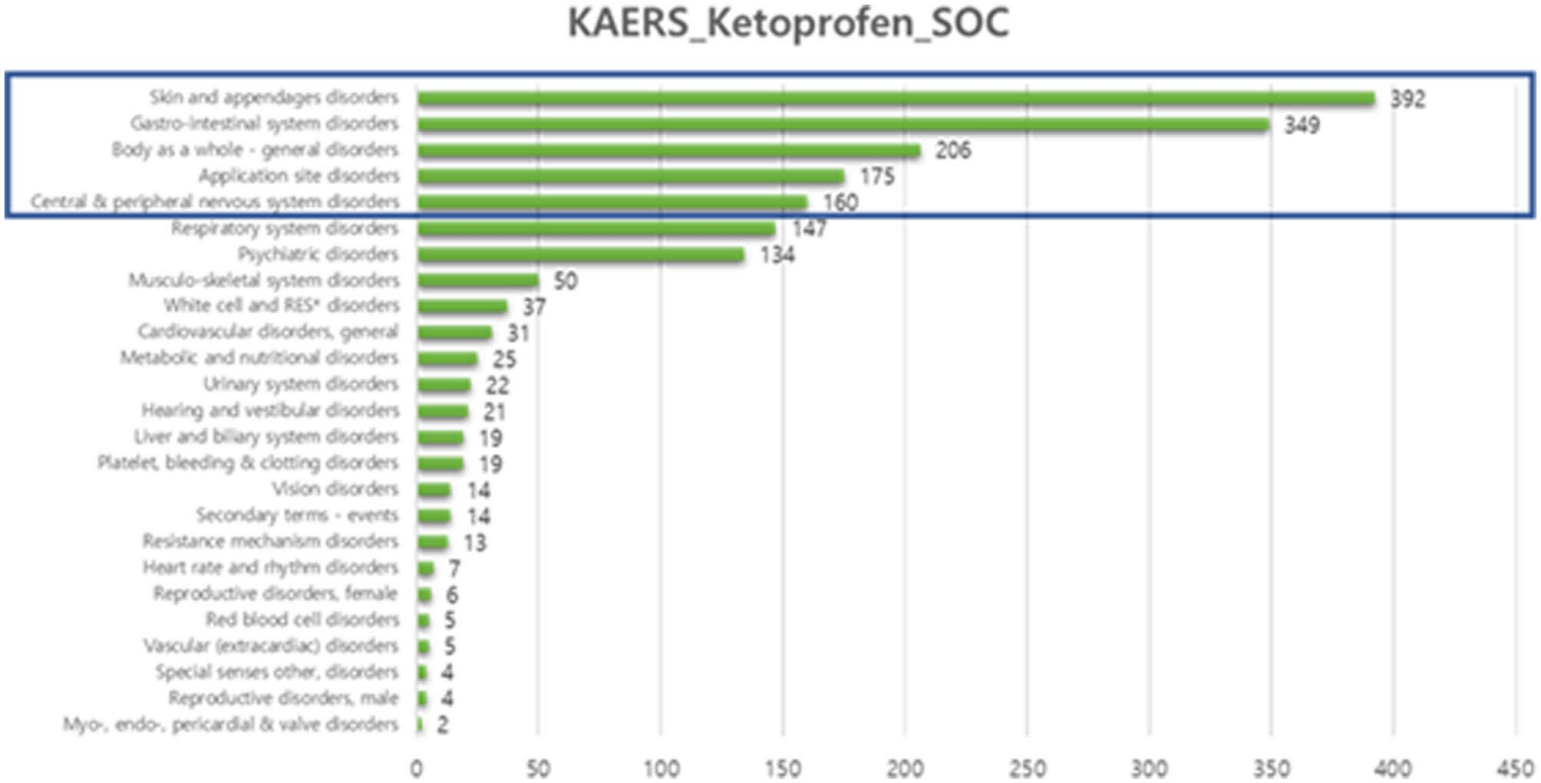
SOC ranking graph for KAERS-based ketoprofen adverse event reporting.

The Korea Adverse Event Reporting System (KAERS) database contains adverse event report dates, reporter information, patient information such as gender and age, the ingredient name, the effect group classification of the suspected drug, and causality evaluation data. KAERS has the advantage of detecting side effects that have not been identified in pre-marketing clinical trials and early detection of adverse reactions that occur rarely after drug marketing. For this, it is a system in which doctors, pharmacists, and patients themselves report drugs taken and suspected adverse events to administrative authorities or related drug monitoring centers^34^. All drug names were coded using the Anatomical Therapeutic Chemical Classification System code. ADRs were coded as WHO–ART Preferred Terms^26^. The SOC rankings of the 960 patients (2013–2017) in KAERS reporting ADRs with ketoprofen followed a similar pattern (Fig. 6). Spontaneously reported KAERS-based and detected SNS-based adverse events resembled the top SOC groups.

## Discussion

The aim of this study was to establish whether side effect data for commercially available drugs can be curated and utilized through social media. Unstructured data processing and relationship analyses are applied to social media data. We investigated whether these modalities can be effectively used for drug surveillance and proposed a novel drug side effects data curation pipeline based on these methods.

The pros and cons of using social media data for pharmacovigilance have been comprehensively reviewed^29^. We explored whether social media analysis could facilitate early detection of unknown adverse events and supplement spontaneous reporting systems. We examined whether it was possible to integrate social media analyses with spontaneous reporting systems to improve ADR signal discovery^27^. We also considered the use of social data. As social media conversations have a broad scope, they might also include health-related topics. Hence, these data could be used to detect potentially novel ADRs with less latency. Progress has been made in research on ADR detection via social media. To the best of our knowledge, however, no prior study has integrated drug safety evidence from the spontaneous reporting system KAERS and the social channel Naver in South Korea.

Here, we assessed the utility of social media data in pharmacovigilance. We exclusively targeted Naver posts but collected data from two channels (Naver blog and café) to augment heterogeneity. (1) We found that the number of articles available online is rapidly increasing for target drugs. Future pharmacovigilance studies could be performed by accumulating sufficient side effects data using SNS data. (2) We proposed a method of constructing a side effects dictionary based on SIDER and WHO–ART mapping and lexicon definition. The latter is vital to South Korean natural language processing. We could expand the side effect dictionary from the perspective of individuals using the drugs inducing the adverse reactions. This lexicon-based exploratory data analysis identified side effect posts among unlabeled ones. (3) The words we sought for the target marketed drug referred to officially published side effects information on it. We confirmed that frequency-based quantitative patterns of side effects obtained via SNS did not differ from the SOC range of self-reported side effect information acquired from KAERS. Hence, our research results were reliable.

The present study had several limitations. First, our information was restricted to the user as we only collected data from Naver posts. Other social channels such as Twitter were not considered here. Second, when there was no indication whether a post found by crawling was, in fact, a valid article, we executed the selection based on key words. In future research, it will be necessary to improve the search and valid post selection methods. Third, we only defined known side effects by constituting the Lexicon. If side effects are also defined by extension of the Lexicon to detect indications and unknown side effects, postings with more diverse characteristics may also be explored. Fourth, there is insufficient evidence to demonstrate that postings deemed valid and explaining side effects are clinically relevant. More meaningful interpretations might be achieved through ongoing consultation with clinical experts. Finally, you will have to think about the users of the crawled dataset. We tried to secure data suitable for the target, even considering the age bias of SNS users, because the users of pharmacovigilance monitoring data for the elderly include not only the elderly but also the families who actually support the elderly. Fortunately, the elderly in Korea have a significantly higher smartphone ownership rate compared to other smart devices^32^, and those aged 65 and over have the highest smartphone ownership rate in the world^33^. However, we are still concerned that we will have to conduct various comparative studies on whether our dataset is the most optimal.

We reviewed validation studies of ketoprofen using well-known side effect information. In the future, the study will not be limited to well-known side effects and will study unknown side effects while expanding this study to drugs with greater demand, such as Tylenol and Aspirin. In addition, we intend to additionally apply various machine learning techniques. The newly derived information can be used as data that can be used to make recommendations for policy establishment by actively utilizing the consumer's point of view of drug use.

## Conclusion

In the present study, we proposed a standard analytical pipeline for monitoring drug side effects using SNS data. We then informatically validated this tool using a prescription drug commonly prescribed to elderly patients. The pipeline could identify the known ADR symptoms and compile information on co-administered drugs from SNS data. Based on the drug information alone, it was confirmed that drug side effects may be monitored according to the SNS data and from the perspective of consumers. Thus, SNS data can also be used to search for ADR information and identify the characteristics of patients presenting with ADR. Furthermore, SNS data could also support data post labeling for AI learning.

## Data Availability

Crawled Social Media data not included in the manuscript. Derived data supporting the findings of this study, word embeddings and text mining data, are available to the corresponding authors upon request.

## Abbreviations

ADR: Adverse drug reaction
API: Application programming interface
KAERS: Korea adverse event reporting system
Kiwi: Korean intelligent word identifier
KPIS: Korea pharmaceutical information service
MedDRA: Medical dictionary for regulatory activities
SIDER: Side effect resource
SNS: Social network study
SOC: System organ class
URL: Uniform resource locator
WHO–ART: World health organization adverse reaction terminology
